# How do psychology researchers interpret the results of multiple replication studies?

**DOI:** 10.3758/s13423-022-02235-5

**Published:** 2023-01-12

**Authors:** Olmo R. van den Akker, Jelte M. Wicherts, Linda Dominguez Alvarez, Marjan Bakker, Marcel A. L. M. van Assen

**Affiliations:** 1 Department of Methodology and Statistics, Tilburg University, Warandelaan 2, 5037 AB Tilburg, The Netherlands; 2 Department of Sociology, Utrecht University, Utrecht, The Netherlands

**Keywords:** Multi-study paper, Replication, Statistical misinterpretation, Heuristics, Bayesian inference, Vote counting

## Abstract

Employing two vignette studies, we examined how psychology researchers interpret the results of a set of four experiments that all test a given theory. In both studies, we found that participants’ belief in the theory increased with the number of statistically significant results, and that the result of a direct replication had a stronger effect on belief in the theory than the result of a conceptual replication. In Study 2, we additionally found that participants’ belief in the theory was lower when they assumed the presence of *p*-hacking, but that belief in the theory did not differ between preregistered and non-preregistered replication studies. In analyses of individual participant data from both studies, we examined the heuristics academics use to interpret the results of four experiments. Only a small proportion (Study 1: 1.6%; Study 2: 2.2%) of participants used the normative method of Bayesian inference, whereas many of the participants’ responses were in line with generally dismissed and problematic vote-counting approaches. Our studies demonstrate that many psychology researchers overestimate the evidence in favor of a theory if one or more results from a set of replication studies are statistically significant, highlighting the need for better statistical education.

## Introduction

Imagine the following situation: you have conducted four psychology experiments that all tested a given theory. All four experiments had a power of 50% and two out of the four experiments yielded statistically significant results. Assuming that your belief in the validity of the theory before conducting these experiments was 50%, what would your current belief in the theory be? Given that the contemporary psychology literature mainly includes statistically significant results (Fanelli, 2010, 2012; Hartgerink et al., 2016; Sterling et al., 1995), one might think the theory is valid only when all experiments yielded significant results. However, this would be mistaken. Using Bayes’ rule, we can calculate that the probability of the theory being correct when two out of four results are significant is as high as 97% (see Box 1). Based on the wealth of studies that show that academics often have trouble with correctly interpreting statistical results (Aczel et al., 2018; Fischhoff et al., 1983; Gigerenzer, 2018; Hoekstra et al., 2006, 2014; Kahneman et al., 1973), we suspect that this result would surprise many readers.

**Box 1 – Assessing belief in the theory using Bayes’ theorem**

**Table:** 

The validity of a theory (*H*_*A*_) given multiple (non)significant experiments (i.e., the probability that the theory is correct given the data) depends on the power of the experiments and can be readily computed with Bayes’ theorem. Formally: $$P\left(H_Ais\;true\right|data)=\frac{P\left(H_Ais\;true\cap data\right)}{P\left(data\right)}=\frac{P\left(data\vert H_Ais\;true\right)\ast P\left(H_Ais\;true\right)}{P\left(data\vert H_Ais\;true\right)\ast P\left(H_Ais\;true\right)+P\left(data\vert H_0is\;true\right)\ast P\left(H_0is\;true\right)}=\frac{\begin{pmatrix}N\\k\end{pmatrix}\ast\left(1-\beta\right)^k\ast\beta^{\left(N-k\right)}\ast P\left(H_Ais\;true\right)}{\begin{pmatrix}N\\k\end{pmatrix}\ast\left(1-\beta\right)^k\ast\beta^{\left(N-k\right)}\ast P\left(H_Ais\;true\right)+\begin{pmatrix}N\\k\end{pmatrix}\ast\alpha^k\ast{(1-\alpha)}^{\left(N-k\right)}\ast P\left(H_0is\;true\right)}$$ If we assume that $P(H_Ais\;true)=P(H_0is\;true)=0.5$ these terms drop out. And since $$\left(\begin{array}{c}N\\ k\end{array}\right)$$ also drops out, we obtain: $p\left({H}_{A}|k\right)=\frac{{\left(1-\beta \right)}^{k}*{\beta }^{\left(N-k\right)}}{{\left(1-\beta \right)}^{k}*{\beta }^{\left(N-k\right)}+{\alpha }^{k}*{(1-\alpha )}^{\left(N-k\right)}}$ (1) where (1-β) is power, α is the significance level, *N* is the total number of experiments, and *k* is the number of statistically significant results. For our example in the *Introduction* with a power of 0.50, a significance level of 0.05, and two out of four significant results, the probability that the theory is correct is 0.965 (see Eq. 2) $p\left({H}_{A}|2\right)=\frac{{\left(1-0.50\right)}^{2}*{0.50}^{\left(4-2\right)}}{{\left(1-0.50\right)}^{2}*{0.50}^{\left(4-2\right)}+{0.05}^{2}*{(1-0.05)}^{\left(4-2\right)}}\approx 0.965$ (2) When only one out of four results is significant, and using the same values for $\alpha$ and $\beta$, the probability that the theory is correct is still 0.593. In case of a statistical power of 0.80, the posterior belief in the theory is lower than when power is 0.50 for zero, one, and two statistically significant results (see Fig. 1) **Fig. 1:** 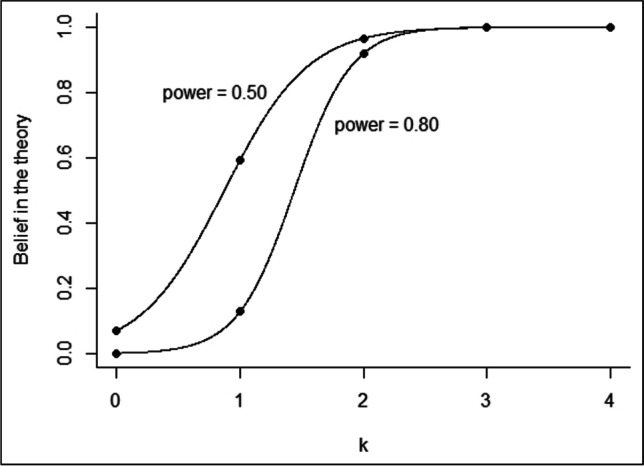 Belief in the theory based on Bayesian inference, as a function of statistical power (0.50 and 0.80) and the number of statistically significant results, *k*, given prior probabilities equal to 0.5. The beliefs in the theory for *k* = 0,1,2,3,4 are [0.071, 0.593, 0.965, 0.998, 0.999] and [0.002, 0.013, 0.919, 0.999, 1.000] for a statistical power of 0.50 and 0.80, respectively	

We carried out two between-subjects vignette studies to test academics’ statistical intuitions when assessing the results of multiple (four) experiments. We decided to carry out Study 2 in 2022 because Study 1 was conducted in 2014 and a lot has changed in the meantime. For example, *p*-hacking (Friese & Frankenbach, 2020; Head et al., 2015; Wicherts et al., 2016) and the statistical interpretation of replication studies (Klein et al., 2018; Maxwell et al., 2015) have been discussed widely, and numerous educational materials appeared on these topics (Azevedo et al., 2019; Da Silva Frost & Ledgerwood, 2020). Increased awareness about these issues raised the question whether the results of Study 1 are still relevant to how researchers today think about the results of replication experiments. Below we will first outline the research questions that were common to both studies and then outline the research questions that were unique to each study.

To examine the relationship between belief in the theory and the number of statistically significant results, in both studies we varied the number of significant results from zero to four, out of four experiments. We expected a positive relationship but we did not have any predictions about the nature of this relationship (e.g., linear, quadratic, etc.). To uncover whether different types of replications differentially affect belief in the theory, we also presented the experiments as being either a direct or a conceptual replication. We expected academics to evaluate a significant conceptual replication as providing more evidence for the validity of the theory than a significant direct replication. We based this prediction on the strong focus on novelty and generalizability in academia, where academics might find a replication using different methods or designs more convincing (Crandall & Sherman, 2016; Giner-Sorolla, 2012; Schmidt, 2009).

In both studies, we used Bayes’ rule to calculate participants’ accuracy: how well their stated belief in the theory corresponded to the correct computation according to Bayes’ theorem (see Box 1). Assuming that experience and knowledge positively predict the accuracy of participants’ posterior beliefs, we expected a positive association between accuracy and participants’ number of peer-reviewed publications and (self-reported) statistical knowledge.

In Study 1 only, we randomly allocated participants to the role of “author” or “reviewer” to examine if authors and reviewers differ in their assessment of the set of results. Specifically, we asked participants in these roles two questions: (1) if they would submit the set of results to a journal (author) or recommend it for publication (reviewer), and (2) whether they would run an additional replication experiment before possibly submitting (author) or whether they would demand that the authors to carry out an additional replication experiment (reviewer). We did not have expectations regarding these questions.

In Study 2 only, we included a regular condition and a preregistration condition. The regular condition was equivalent to the vignette of Study 1 in that the replication studies were said to be typical for psychology. In the preregistration condition, the replication studies were said to be preregistered and aligned with their preregistrations. Thus, the two conditions would differ in the degree to which *p*-hacking could have occurred. *P*-hacking involves collecting or selecting data or analyses to render nonsignificant results significant (Head et al., 2015) and may lead to false-positive results (i.e., results that are an artefact of the researcher’s decisions instead of evidence in favor of an underlying theory). We therefore expected that the participants in the regular condition would have a lower belief in the theory than in the preregistration condition when confronted with significant results. In Study 2 only, we also explicitly asked whether participants considered *p*-hacking when assessing belief in the theory. For scenarios with statistically significant results, we expected participants who considered the possibility of *p*-hacking to show lower belief in the theory than participants who did not.

Finally, using individual participant data from both studies, we sought to categorize participants’ assessments of the results of the four experiments into several heuristics used to weigh the evidence. We now present the methods and results of Study 1 and Study 2, and then provide more information about the Heuristic Analyses.

## Method of Study 1

### Sample selection

We sampled participants from social and experimental psychology who commonly conduct (as researcher) or judge (as editor) experimental research consisting of multiple studies. In both social and experimental psychology, a single study is typically not considered to be sufficient to test a theory (Murayama et al., 2013), and multiple study papers are the norm (Giner-Sorolla, 2012). Using Web of Science, we selected empirical journals in social and experimental psychology published in English that had a 5-year Impact Factor higher than 2 in the year 2012. From social psychology, we included *Journal of Personality and Social Psychology, Journal of Experimental Social Psychology, Personality and Social Psychology Bulletin,* and *European Journal of Social Psychology*. From experimental psychology, we included *Journal of Experimental Psychology – General, Journal of Experimental Psychology – Human Perception and Performance, Journal of Experimental Psychology – Learning, Memory, and Cognition, Quarterly Journal of Experimental Psychology,* and *Cognition & Emotion*. In total, we collected 2,449 references to articles published in 2012 and 2013. We included one additional journal for the subfield of experimental psychology to keep the number of articles between the subfields approximately equal, resulting in 1,126 articles for social psychology and 1,323 articles for experimental psychology.

To contact researchers, we retrieved contact information of the corresponding authors from Web of Science. After deleting duplicate email addresses, we ended up with 1,810 unique researchers. To contact editors, we looked up the editorial board of the selected journals and searched online for the contact details of the represented (associate) editors and reviewers, yielding contact details for 834 unique editors. Of the 2,644 potential participants of Study 1 (1,322 in each assigned role), 52 emails proved invalid, so only the remaining 2,592 researchers and editors received an invitation to participate in the survey. The invitations were sent at the end of May and the beginning of June 2014. We sent a reminder 2 weeks later and stopped collecting data 2 weeks after the reminder. After excluding the non-completers, 228 academics participated in the authors’ version and 277 academics in the reviewers’ version, with response rates of 17.6% and 21.4%, respectively.

### Procedure and materials

We used Qualtrics to conduct the survey for Study 1. Before presenting the survey, the participants in the sample were randomly assigned to the authors’ version (see https://osf.io/aufn2) or to the reviewers’ version (see https://osf.io/hqx4e) of the survey. The study involved eight different scenarios, each presenting the results of four experiments. All presented scenarios stated that other researchers had previously published the results of one experiment, A, and found a statistically significant effect in line with a given theory. The vignette then stated that the participant had conducted (“authors”) or was asked to review (“reviewers”) four experiments that replicated the findings of the original study. The first new experiment (A’) was a direct replication of the earlier experiment, whereas the other three experiments (B, C, and D) were conceptual replications. Participants were presented with four out of eight possible scenarios in Table 1, where each scenario had a different number of significant results, *k*. All participants were told to imagine that their prior belief in the theory before seeing the results of the four experiments was 50%, and that the number of participants, the costs of all experiments, the nominal significance level, and the statistical power in all five experiments (including the original experiment A) were typical for experimental studies in psychology.

**Table 1:** Summary of the eight different scenarios presented in the vignettes of Study 1 and Study 2

Scenario	A’	B	C	D	*k*
1	O	O	O	O	0
2	X	O	O	O	1
3	O	X	O	O	1
4	X	O	X	O	2
5	O	X	X	O	2
6	X	O	X	X	3
7	O	X	X	X	3
8	X	X	X	X	4

X indicates significant results, whereas O indicates non-significant results. A’ indicates a direct replication, whereas the remaining letters indicate conceptual replications. *k* refers to the number of statistical results in each scenario

After providing informed consent, participants read the introduction stating that a distinction was made between direct replications and conceptual replications. We clarified that a direct replication uses the same method as the original study and tries to reproduce it as closely as possible, while a conceptual replication may use different methods or operationalizations (Schmidt, 2009). Next, participants were successively shown a table for each of the scenarios. Those tables included information about which of the experiments showed a statistically significant result and were shown at the top of every page, preventing participants from forgetting the results in the scenario (see Table 1 for all eight possible scenarios). In six scenarios (those with 1–3 significant results) either A’ or B was significant. For instance, Scenarios 2 and 3 both have one significant experimental result, but in Scenario 2 study A’ (direct replication) is significant and in Scenario 3 study B (conceptual replication) is significant. Participants were randomly assigned to either Scenario 2 or Scenario 3 (both with *k* = 1), either Scenario 4 or Scenario 5 (both with *k* = 2), and either Scenario 6 or Scenario 7 (both with *k* = *3)*. In addition, participants were randomly assigned to either Scenario 1 (with *k* = 0) or Scenario 8 (with *k* = 4). Participants thus considered four scenarios in total.

After each scenario, participants indicated their belief in the theory on the basis of the presented evidence by means of a slider bar, with points going from low probability (0%) to high probability (100%) of the theory being correct. Participants could indicate using a text box whether they missed any information while reading the scenarios. Next, they had to indicate the statistical power, ranging between 0 and 1, they had in mind while answering the questions.^1^ The survey ended with four demographic and work-related questions: gender, year that they obtained their doctorate, number of peer-reviewed papers published (using six categories: < 5, 5–15, 16–30, 31–50, 51–100, and > 100), and the participant’s self-reported statistical knowledge on a scale from 0 (poor) to 10 (excellent). Finally, we gave participants the option to write down any comments regarding the survey or research project and we thanked them for their participation. The responses of all participants of Study 1 can be found at https://osf.io/k4us3.

To assess participants’ accuracy in estimating the probability that the theory is correct, we created an accuracy variable; for every *k* in every different scenario, we calculated the root mean squared error (RMSE) comparing the participant’s belief in the theory with the normative Bayesian prediction (see Eq. 1, Box 1):

3 $$RMSE=\sqrt{\frac{1}{j}{{\sum }_{i=1}^{j}({m}_{i}-{d}_{i})}^{2}}$$

where *j* refers to the number of responses of a participant (typically 4), *m* refers to the responses as predicted by Bayesian inference, and *d* refers to the beliefs stated by the participants.

In the Qualtrics survey of Study 1, we also included a question reagrding whether participants would submit (as author) or accept (as reviewer) a set of studies for publication, a question regarding whether they would require an additional experiment, and a dichotomous question about their belief in the theory. Due to space constraints, we do not present the results related to these questions in this paper but interested readers can find them at https://osf.io/vnws7.

## Method of Study 2

### Sample selection

For Study 2 we searched the Web of Science Core Collection for journal articles from the research areas social psychology and experimental psychology published in the years 2020 (searched on 8 February 2021) and 2021 (searched on 3 December 2021). We did not include any papers published before the 2020s because researchers’ ideas about open science and statistical inference seem to be changing rapidly and we wanted to be able to draw conclusions about the current timeframe. Our search yielded 14,940 (for the year 2020) + 14,480 (for the year 2021) references, each with one email address. After removing duplicates, we were left with 21,120 unique email addresses. 2,632 of those were out of office, while 794 emails proved invalid. The remaining 19,694 researchers received an invitation to participate in the week of 14 February 2022. Those who did not reply received a reminder 2 weeks later. We stopped data collection on 25 May 2022. In total, 1,334 researchers participated in Study 2, equally distributed over the preregistration condition and the regular condition. The response rate was 6.8%.

### Procedure and materials

We again used Qualtrics to develop the survey for Study 2 (see https://osf.io/xycte for the full survey). Some participants of Study 1 indicated in open comments that the survey questions were difficult to answer because the vignette lacked detailed information. Notably, 22 participants (4.4%) expressed confusion about the role of statistical power in our experiment. To prevent any issues, we provided more specific information in Study 2 as we stated that the significance level (= 0.05) and statistical power if the theory is valid (= 0.50) were the same for each experiment (A, A’, B, C, and D). This seemed to have helped as only 14 participants (1.0%) expressed confusion about statistical power in Study 2. We also emphasized in the vignette of Study 2 that the prior belief of 50/50 pertains to the situation *after* seeing the result of the original experiment, but *before* seeing the results of the replication experiments.

The main change from Study 1 was that we randomly assigned Study 2 participants to a preregistration condition or a regular condition. In the preregistration condition participants were told that the design and analysis of all replication experiments were preregistered and that they were conducted and analyzed in line with their preregistrations. In the regular condition (corresponding to the author vignette of Study 1), participants were told to assume that the replication experiments were typical for experimental studies in psychology. We also explicitly asked about *p*-hacking: “Throughout this study, did you consider the possibility that the researcher in the scenarios made decisions through which they, consciously or subconsciously, directed their experiments toward a statistically significant result?” The responses of the participants of Study 2 can be found at https://osf.io/5dfhs.

The design, hypotheses, and statistical analyses for Study 2 were preregistered (see https://osf.io/f7vsq). Hypotheses 1 through 4 (regarding the number of statistically significant results, replication status, the number of publications, and statistical knowledge) were limited to participants in the preregistration condition to make comparison with Study 1 possible. Hypotheses 5 and 6 (regarding preregistration status, and the possibility of *p*-hacking) were related to all participants. For completeness we also ran the first four hypotheses using all participants (see https://osf.io/f7ymv for the results of these analyses).

## Results of Study 1 and Study 2

All analyses were carried out using R version 4.1.2. The R-code used for the analyses can be found at https://osf.io/jq3w7 (Study 1) and https://osf.io/4cx6w (Study 2).

Table 2 shows the average belief in the theory (0–100%) for each number of statistically significant results, and the composition of significant results (direct or conceptual replication significant) for Studies 1 and 2. We used multilevel linear regression to test the hypothesized association between the number of significant results and belief in the theory. The dependent variable was a logit transformation of belief divided by 100, which makes the effective relationship between the independent variables and belief non-linear, while preserving the linear model. As expected, average belief increased with the number of statistically significant results (Study 1: $\beta$ = 0.74, 95% CI = [0.69, 0.80], *p* < 0.001; Study 2: $\beta$ = 0.74, 95% CI = [0.69, 0.79], *p* < 0.001).

**Table 2:** Mean (standard deviation) of belief in the theory in percentages, for every number of significant results, *k*, and for the different compositions of statistical results

	Participants’ mean belief in Study 1	Participants’ mean belief per composition in Study 1		Participants’ mean belief in Study 2	Participants’ mean belief per composition in Study 2	
		A’ significant	B significant		A’ significant	B significant
*k* = 0	25.33 (16.83)	-	-	23.98 (18.50)	-	-
*k* = 1	33.04 (17.14)	33.93 (17.82)	32.15 (16.42)	33.51 (19.04)	34.36 (20.35)	32.75 (18.13)
*k* = 2	49.34 (15.27)	50.65 (15.27)	48.00 (15.19)	46.67 (17.52)	48.65 (17.49)	43.62 (18.06)
*k* = 3	65.77 (16.18)	67.13 (16.11)	64.38 (16.16)	61.15 (18.77)	64.16 (17.16)	57.78 (19.86)
*k* = 4	73.02 (17.65)	-	-	71.08 (18.47)	-	-

*k* refers to the number of significant results within the scenario. ‘A’ significant’ means that the direct replication was significant, ‘B significant’ means that the conceptual replication was significant

Unexpectedly, in both studies, participants’ average beliefs in the theory were higher when the *direct* replication was significant than when the *conceptual* replication was significant (Study 1: $\beta$ = -0.13, 95% CI = [-0.21, -0.05], *p* = 0.0017; Study 2: $\beta$ = -0.23, 95% CI = [-0.31, -0.15], *p* < 0.001). Average belief in the theory was between 1.78% points (*k* = 1) and 2.75% points (*k* = 3) higher for direct than for conceptual replications in Study 1, and between 1.61% points (*k* = 1) and 6.38% points (*k* = 3) higher in Study 2 (see Table 2).

For our next hypotheses, we measured the accuracy of participants’ posterior beliefs by comparing their responses to the responses predicted by Bayesian inference. In Study 1 we based this on the participants’ reported power, thereby excluding participants who did not provide a power level. In Study 2 we used a power of 0.5 as this was the power level disclosed in the vignette. We regressed participants’ root mean squared error (see Eq. 2) on Papers published^2^ and Statistical knowledge, and found number of published papers to linearly predict higher accuracy in Study 1 ($\beta$= 0.0057, 95% CI = [0.0021, 0.0093], *p* = 0.0019), but found no such association in Study 2 ($\beta$= -0.0002, 95% CI = [-0.0004, 0.000002], *p* = 0.0537). For statistical knowledge, we found a nonsignificant association in both Study 1 ($\beta$= -0.0034, 95% CI = [-0.007, 0.00015, *p* = 0.061) and Study 2 ($\beta$= 0.008, 95% CI = [0.002, 0.014], *p* = 0.0135).

In Study 2, we also looked at the influence of preregistration status and the possible presence of *p*-hacking. Contrary to our hypothesis, we found no difference between the preregistration condition and the regular condition, $\beta$ = 0.02, 95% CI = [-0.10, 0.13], *p* = 0.783 (Model 2C in Table 3). However, as expected, we found that participants had lower beliefs in the theory when they considered the researcher in the vignette to have used *p*-hacking ($\beta$ = -0.31, 95% CI = [-0.43, -0.20], *p* < 0.001 (Model 2D in Table 3). As a manipulation check, we also checked whether participants more often took into account *p*-hacking in the regular condition (38.2%) than in the preregistration condition (33.9%). This was indeed the case, *t*(4177.6) = 2.93, *p* = 0.003.

**Table 3:** Fixed effects estimates (top) and variance estimates (bottom) for the multilevel linear regression of belief in the theory on the number of significant results, the composition of significant results, preregistration status, and the possible presence of *p*-hacking – for all participants in Study 1 (Models 1a and 1b), only participants in the preregistration condition in Study 2 (Models 2a and 2b), and all participants in Study 2 (Models 2c and 2d)

Parameters	Model 1A	Model 1B: 1A + Conceptual	Model 2A	Model 2B: 2A + Conceptual	Model 2C: 2A + Preregistration	Model 2D: 2A + *p*-hacking
*Regression coefficients (fixed effects)*						
Intercept	-1.63 (0.074) *	-1.57 (0.076) *	-1.67 (0.07) *	-1.45 (0.075) *	-1.62 (0.06) *	-1.49 (0.06) *
Level 1						
* k*	0.74 (0.028) *	0.74 (0.028) *	0.74 (0.26) *	0.69 (0.03) *	0.71 (0.02) *	0.72 (0.02) *
Conceptual	-	-0.13 (0.041) *	-	-0.23 (0.04) *	-	-
Preregistration	-	-	-	-	0.02 (0.06)	-
* p*-hacking	-	-	-	-	-	-0.31 (0.06) *
*Variance components (random effects)*						
Residual	0.44 (0.66)	0.43 (0.65)	0.84 (0.92)	0.48 (0.70)	0.66 (0.81)	0.58 (0.76)
Intercept	2.15 (1.47)	2.15 (1.47)	2.03 (1.43)	2.42 (1.55)	2.07 (1.44)	2.06 (1.43)
Slope	0.28 (0.53)	0.28 (0.53)	0.30 (0.55)	0.34 (0.58)	0.23 (0.47)	0.24 (0.49)
*r(intercept, slope)*	-0.78	-0.78	-0.74	-0.73	-0.75	-0.77

Standard errors are in parentheses. *k* refers to the number of significant results within the scenario. Conceptual is a binary variable that takes on the value of 1 if the conceptual replication was significant and 0 otherwise. Preregistration is a binary variable that takes on the value of 1 if the participant was allocated to the preregistration condition and 0 if the participant was allocated to the regular condition. *p*-hacking is a binary variable that takes on the value of 1 if the participant indicated to have taken into consideration *p*-hacking in their responses and 0 if they did not indicate this

^*^ *p* $$<$$ .001

## Heuristic Analyses

Averaged results of Study 1 aligned with a heuristic where the prior belief of 50% is averaged with the percentage of statistically significant results (see Table 2). However, the results also indicated that participants varied in how *k* affected their beliefs (i.e., multilevel analyses highlighted a random slope). Therefore, we decided to analyze the data for each participant to find out which heuristics may have been used by *individual* academics. We did this for the data of both studies.

## Method of the Heuristic Analyses

The analyses of individual participant data were preregistered on 19 May 2018 (Study 1: https://osf.io/hjkpx) and 16 February 2022 (Study 2: https://osf.io/f7vsq). To allow accurate preregistration for Study 1, a research assistant blinded the data using a blinding protocol, R-code, and mock data can be found in the folder named “Mock Data for Study 1” at https://osf.io/2g4wf.

**Fig. 2:**
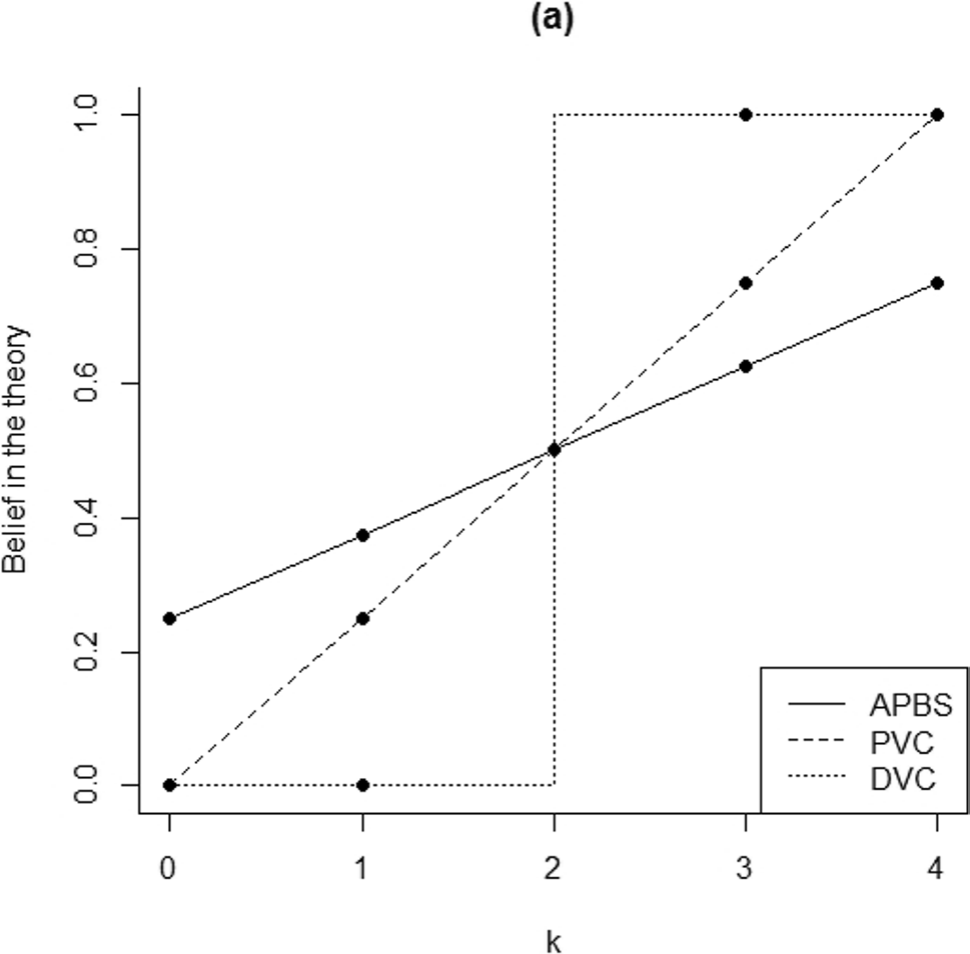
An overview of the three non-normative heuristics that are potentially being used. “APBS” refers to averaging prior belief and significance, “PVC” refers to proportional vote counting, and “DVC” refers to deterministic vote counting

In addition to Bayesian inference (Box 1), we considered three potential heuristics that academics may have used when interpreting the outcomes of multiple experiments. We label these heuristics “deterministic vote counting,” “proportional vote counting,” and “averaging prior belief with significance.” The predictions of these three heuristics are shown in Fig. 2. For simplicity’s sake, none of the heuristics take into account the participants’ assigned role (relevant to Study 1 only) nor the type of replication (relevant to both studies).

Deterministic vote counting and proportional vote counting are based on the possibility that academics interpret null hypothesis significance test results dichotomously (Hoekstra et al., 2006; Rosenthal & Gaito, 1963, 1964) and therefore weigh the evidence by counting the number of (non-)significant outcomes in a set of studies (Hedges & Olkin, 1980). Deterministic vote counting occurs when researchers believe the theory is true if the proportion of significant results exceeds 0.5, will believe the theory is false when that proportion is below 0.5, and have a 50/50 belief if the proportion equals 0.5. Academics employing proportional vote counting equate their belief in the theory to the proportion of significant results. Finally, when academics employ the heuristic of averaging, they simply average their prior belief in the theory before seeing the results of the four experiments (50/50 in our scenarios) with the proportion of significant results. The three heuristics are analyzed together with the normative Bayesian inference approach outlined in Box 1.

Adhering to our preregistration, we discarded participants whose belief in the theory showed no (weakly) monotonic increase in the number of significant results (Study 1: N = 70, 13.9%; Study 2: N = 329, 24.7%), participants with three or four (out of four) missing values on the belief variable (N = 47, 9.3%; only relevant for Study 1), and/or participants with a missing value on self-assessed power (N = 1272, 24.2%; only relevant for Study 1). The remaining sample for Study 1 involved 312 participants, of whom 152 were presented the author’s version and 160 the reviewer’s version. The remaining sample for Study 2 involved 1,005 participants, of whom 493 were allocated to the preregistration condition and 512 to the regular condition.

The remaining responses were analyzed using a Bayesian model in which we calculated participants’ posterior probabilities of using one of the four heuristic models (averaging prior belief and significance, proportional vote counting, deterministic vote counting, or Bayesian inference) given their responses, with prior model probabilities equal to 0.25 for each of the four models. In our “weak” classification we allocate a participant to the model with the highest posterior probability (at least 0.25). In our “strong” classification we allocate a participant to a model if their posterior probability for the model exceeds 0.75, which corresponds to a Bayes factor of at least 3.

The posterior probability of a participant using model *H*_*i*_ given the data *X* = {*x*_*1*_, …, *x*_*4*_} is calculated as:

4 $$P\left({H}_{i}|X\right)=\frac{P(X|{H}_{i})}{P(X|{H}_{1})+P(X|{H}_{2})+P(X|{H}_{3})+P(X|{H}_{4})}$$

assuming a uniform prior (P(*H*_*j*_) = 0.25), and P(*X*|*H*_*j*_) denoting the likelihood of the data *X* (four responses) given model *H*_*j*_. The likelihood of each response given a model is a normal density with mean μ as determined by that model and standard deviation σ, truncated at 0 and 1. The standard deviation σ reflects the “random decision error” of participants. In our analysis, we used two levels of random decision error, σ = 0.10 and σ = *q*, where *q* was derived by taking each participant’s lowest RMSE (root mean squared error) out of the four RMSE values (one for each model) and taking the average across all participants of those minimum values. Hence, the value of σ = *q* signifies the average misfit of participants with their best-fitting model. We chose a value of σ = 0.10 a priori based on our own statistical intuitions. More details about this procedure can be found in the preregistrations of these analyses at https://osf.io/hjkpx (Study 1) and https://osf.io/f7vsq (Study 2).

To avoid participants being classified into a heuristic while their response pattern does not fit well with any of the models, we also compared participants’ response patterns against a benchmark heuristic. This benchmark heuristic is the participant’s belief averaged across conditions, or simply a horizontal line corresponding to that participant’s average belief. For example, if a participant stated a belief in the theory of 30%, 60%, 70%, and 100% for *k* = 1, 2, 3, 4, respectively, their average belief is 260/4 = 65%. Note that the benchmark heuristic is dependent on the data, unlike any of the other heuristics. We assessed fit using the RMSE (Eq. 2), and only allocated individuals to a model if its RMSE was lower than for the benchmark heuristic. This held for both the weak and the strong classification procedures.

In Study 2 we added an explicit question about the heuristic used by the participants: “We specified four strategies that researchers may use to assess the probability of the theory being correct in the scenarios we presented. Do you consider one of them applicable to your responses throughout this study? If not, please explain what reasoning you did use to arrive at your responses.”

One major alteration was made from the preregistration of the Heuristic Analysis in Study 1 because we mistakenly assumed that participants were told that the statistical power in all studies was 0.5 (as we did state in Study 2). Instead, participants were told that the power of the experiments was typical for psychology experiments. Participants thus had to imagine and report this power value themselves, which influenced the predictions based on Bayesian inference. Because of this oversight we had to calculate those predictions anew. The mean (standard deviation) and mode of self-reported statistical power in Study 1 were 0.67 (0.20) and 0.80, respectively. The post-preregistration analysis code can be found at https://osf.io/q2n7y.

No alterations were made with regard to the preregistration of Study 2.

## Results of the Heuristic Analyses

**Fig. 3:**
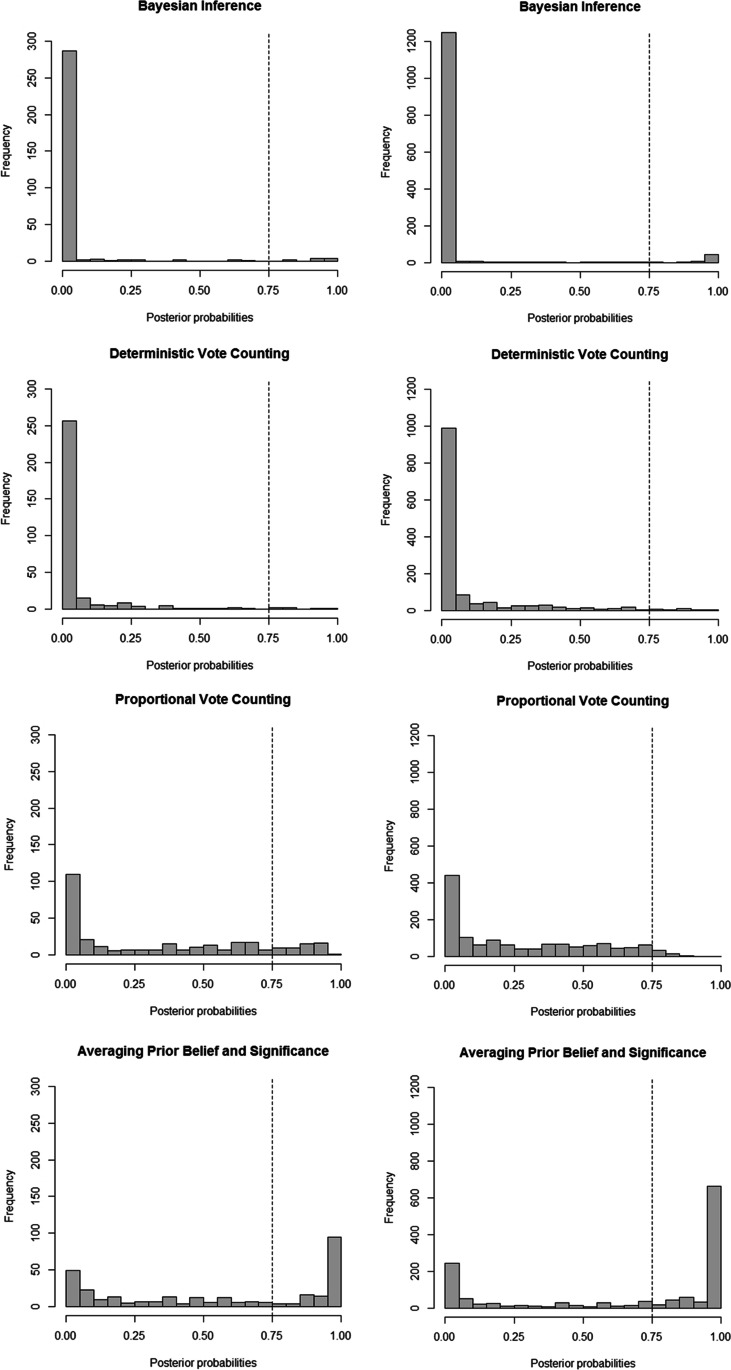
Frequency distributions of the participants’ posterior probabilities of using a model given that they use that model or one of the other models, in the situation where the standard deviation is *q* and where all participants are included (on the left for Study 1, on the right for Study 2). The dotted line represents the threshold value for which that model is three times more likely than the other models combined

The distributions of the analyzed participants’ (*N* = 312 in Study 1 and *N* = 1,005 in Study 2) posterior probabilities of using a particular model are shown in Fig. 3. As evidenced by the low frequency of high posterior probabilities in the upper two panels, only a few participants appear to have used Bayesian inference. In contrast, the high frequency of high posterior probabilities in the bottom two panels of Fig. 3 suggest that many participants averaged their prior belief with the number of significant results.

**Fig. 4:**
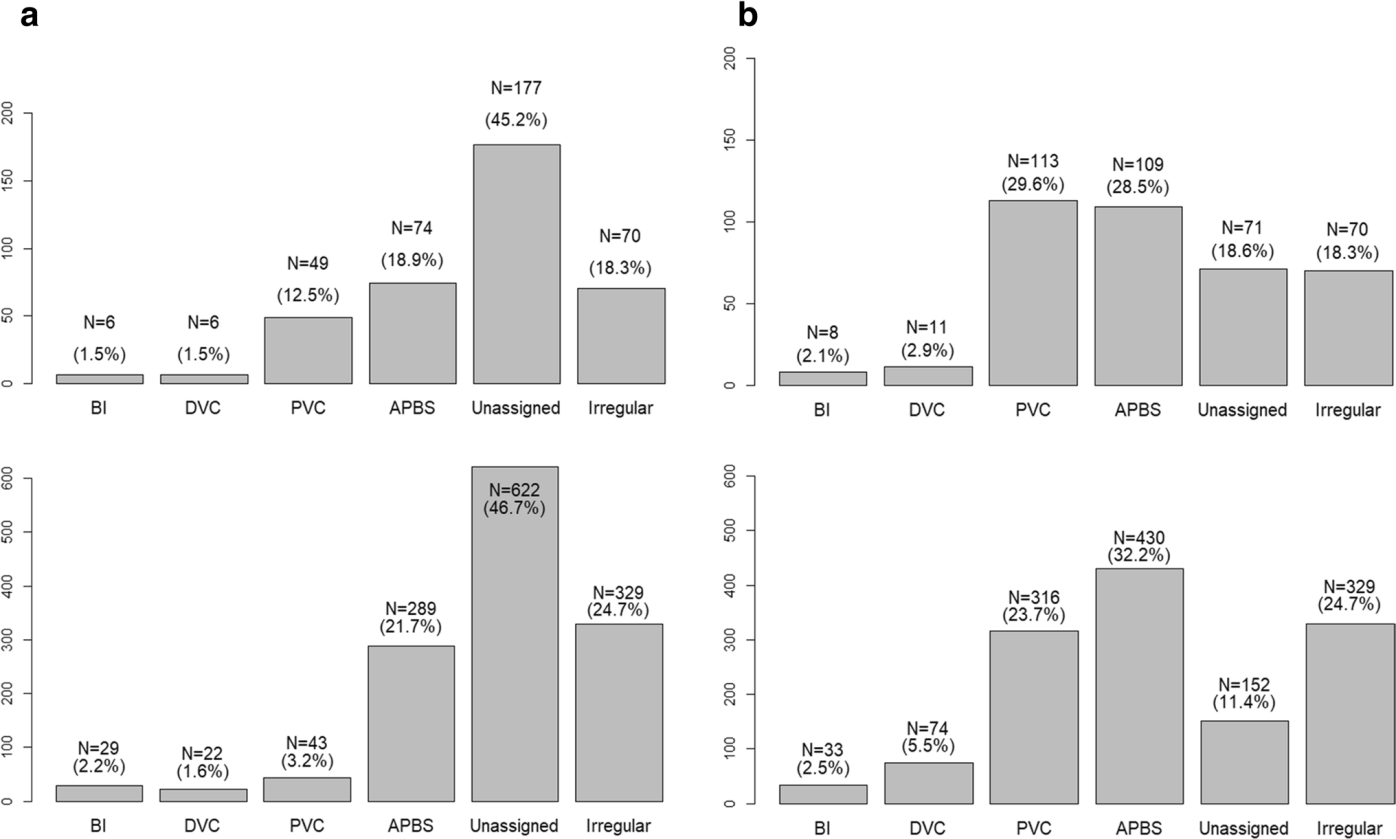
**a** Histogram presenting the strong categorization for Study 1 (top) and Study 2 (bottom). *BI* Bayesian inference, *DVC* deterministic vote counting, *PVC* proportional vote counting, *APBS* averaging prior belief and significance **b** Histogram presenting the weak categorization for Study 1 (top) and Study 2 (bottom). *BI* Bayesian inference, *DVC* deterministic vote counting, *PVC* proportional vote counting, *APBS* averaging prior belief and significance

To assess the robustness of our results to alternative analytic choices, we carried out 3 (participants that faced *k* = 0–3, participants that faced *k* = 1–4, and the whole sample of participants) $\times$ 2 (*q*, the mean of the participants’ lowest RMSEs, and the a priori determined 0.1 as random decision errors) = 6 analyses for Study 1 and two analyses (*q* and 0.1 as random decision errors) for Study 2. For all of the analyses we implemented the weak and strong classification procedure. Because the results of all eight analyses were qualitatively similar (see an overview of all results of Study 1 at https://osf.io/wuje4 and all results of Study 2 at https://osf.io/sw7g5), we decided to only present here the weak and strong classification for the whole sample of participants with σ = *q* = 0.118 (Study 1) and σ = *q* = 0.149 (Study 2). Histograms depicting the number of participants in every category can be found in Fig. 4a (strong categorization) and Fig. 4b (weak categorization).

Relatively few participants used the normative “Bayesian inference” approach (Study 1 – Strong categorization: *N* = 6 (1.6%), Weak categorization: *N* = 8 (2.1%); Study 2 – Strong: N = 29 (2.2%), Weak: N = 33 (2.5%)) and “deterministic vote counting” (Study 1: Strong: *N* = 6 (1.6%), Weak: *N* = 11 (2.9%); Study 2 – Strong: N = 22 (1.6%), Weak: N = 74 (5.5%)). In contrast, a substantial number of participants used “proportional vote counting” (Study 1 – Strong: *N* = 49 (12.5%), Weak: *N* = 113 (29.6%); Study 2 – Strong: N = 43 (3.2%), Weak: N = 316 (23.7%)), and “averaging prior belief and significance” (Study 1 – Strong: *N* = 74 (18.9%), Weak: *N* = 109 (28.5%); Study 2 – Strong: N = 289 (21.7%), Weak: N = 430 (32.2%)). Using strong categorizations, we could not assign 177 participants (45.2%) and 622 (46.7%) participants in Studies 1 and 2, respectively. This occurred because the RMSE for neither heuristic exceeded the RMSE of the benchmark heuristic, or because posterior probabilities of all heuristics were below 0.25. In the weak categorization we could not assign 71 participants (18.6%) in Study1, and could not assign 152 (11.4%) participants in Study 2, because neither heuristic outperformed the benchmark heuristic in RMSE. Finally, in line with our preregistration we also distinguished participants with a response pattern whose belief in the theory did not show an expected (weak) monotonic increase in the number of significant results. Such an “irregular” response pattern was relatively common (Study 1: 70 participants, 18.3%; Study 2: 329 participants, 24.7%).

## Conclusion and discussion

We studied how psychological researchers interpret a set of four replication experiments with varying statistical significance. Across two vignette studies we found that, on average, the number of significant results was positively related to researchers’ belief in the underlying theory. Contrary to our expectations, we found that researchers valued direct replications more than conceptual replications when deciding on the validity of a theory, although this effect was small in both studies. The premium of direct replications over conceptual replications in our studies is surprising in the light of papers that question the importance of direct replications (Cesario, 2014; Schmidt, 2009) and the finding that direct replications are published less often (Makel et al., 2012). It is less surprising in light of the current popularity of large-scale direct replication efforts (Dang et al., 2021; Elliott et al., 2021; Klein et al., 2022). One thing to note when interpreting this result is that people’s judgments tend to be influenced by initially presented values (Furnham & Boo, 2011; Tversky & Kahneman, 1974). Because the direct replication was always listed first in the table outlining the results, this “anchoring effect” could be an alternative explanation for the stronger effect of the direct replication compared to the conceptual replication.

In Study 2, we unexpectedly found that participants’ belief in the theory did not differ when they assessed a set of preregistered versus a set of non-preregistered studies with statistically significant results. Perhaps our manipulation of preregistration was not strong enough, although we found that participants took *p*-hacking into account more often in the regular condition (38.2% of participants) than in the preregistration condition (33.9% of participants), indicating that our manipulation worked at least to some extent. Moreover, participants’ belief in the theory in scenarios with statistically significant results was lower for those who considered *p*-hacking on behalf of the vignette researcher than for those who did not. Combining these findings, we can conclude that psychology researchers are skeptical of statistically significant results when they consider the possibility of *p*-hacking, but that they are also skeptical about the ability of preregistration to effectively prevent *p*-hacking. The latter makes sense in light of findings that preregistrations are not always sufficiently strict to prevent *p*-hacking and are also often not adhered to exactly (Bakker et al., 2020; Van den Akker, 2021).

In the Heuristic Analyses, we zoomed in on individual participant data and categorized participants’ answers into three heuristics and the normative approach of Bayesian inference. Only six out of the 312 analyzed participants (1.6%) in Study 1 and 29 out of 1,334 participants (2.2%) in Study 2 used Bayesian inference, showing that few participants accurately incorporated important parameters like power (1-β) and the significance level (α) into their decisions. Instead, a large proportion of participants (27–33% using our strong categorization, 61% using our weak categorization) used (partial) vote-counting approaches that underestimate the evidence in favor of a theory if two or more out of four results are statistically significant. Additionally, we were not able to categorize a substantial number of participants (45–47% using our strong categorization, 11–19% using our weak categorization), and another group of participants (18–25%) showed an irregular response pattern in which their belief in the theory did not rise with an increase in the number of statistically significant results. Taking these results together, we can conclude that many participants used invalid vote-counting or unknown approaches when interpreting situations with multiple experimental results. Future research could expand on the current study by exploring different heuristics.

A limitation of our study is the stylized nature of the vignette experiments. Indeed, many participants (Study 1: 56.8%; Study 2: 39.0%) expressed that they would prefer to have more information available in the vignette to inform their decisions. This indicates that our results may not accurately map onto real research scenarios. Although we acknowledge that practicing academics may use other available information to ground their beliefs, we were primarily interested in the effects of replication type and preregistration, and therefore designed our vignettes to vary these factors. Future research may examine what other factors affect academics’ belief in a theory. One factor that may be particularly interesting is the number of experiments because including more experiments would make it easier to distinguish the vote-counting rules from Bayesian inference.

Another limitation relates to our method of categorization. We preregistered an elaborate Bayesian method to categorize participants into heuristic categories (see https://osf.io/hjkpx for the preregistration related to Study and https://osf.io/f7vsq for the preregistration related to Study 2), but there are many other ways to do this. To assess the validity of our categorization method, in Study 2 we explicitly asked participants whether they used one of the four heuristics we preregistered. We measured the association between this self-categorization and our own categorizations and found a Cramer’s V of 0.667. This strong association (detailed at https://osf.io/f7ymv) suggests that our method of categorization is largely in line with how the participants themselves thought of their strategies, supporting the validity of our method.

In summary, we found that psychology researchers have poor intuitions when it comes to interpreting a set of mixed experimental replication results. These poor statistical intuitions can lead to the suppression of non-significant findings (publication bias; see Ferguson & Brannick, 2012; Levine et al., 2009), and may lead to inefficient use of resources as both authors and reviewers may require more studies to be run. Moreover, they may lead researchers to engage more frequently in *p*-hacking (John et al., 2012; Simmons et al., 2011; Wicherts, 2017). Poor statistical intuitions not only create incorrect interpretations of experimental results, but also introduce biases in the scientific literature. To avoid this, we need improved education about the interpretation of mixed results. More specifically, we would do well to discourage vote-counting heuristics, which continue to appeal to many yet have been shown to be biased over 40 years ago (e.g., Hedges & Olkin, 1980). Instead, we need to focus our educational efforts on the role that Bayes’ rule plays in statistical inference, possibly combined with teaching how experimental results can be synthesized using meta-analysis. Hopefully, this new focus will result in a less biased scientific literature and fairer judgments about the validity of scientific theories.

## Open Practices Statement

The data, materials, and code for Study 1, Study 2, and the Heuristic Analyses are available at https://osf.io/2g4wf. The preregistrations of Study 2 and the Heuristic Analyses can also be found there.

## Footnotes

1 Note that participants had to provide statistical power themselves because we did not explicitly present them with the statistical power of the experiments in the vignette. In Study 2, we did provide this information.

2 Even though the variable ‘Papers published’ was ordinal and not continuous in Study 1, a plot of the predicted values and residuals indicated no reason to suspect that the linearity assumption was violated.
