# Correction: How do psychology researchers interpret the results of multiple replication studies?

**DOI:** 10.3758/s13423-025-02687-5

**Published:** 2025-04-07

**Authors:** Olmo R. van den Akker, Jelte M. Wicherts, Linda Dominguez Alvarez, Marjan Bakker, Marcel A. L. M. van Assen

**Affiliations:** 1 Department of Methodology and Statistics, Tilburg University, Warandelaan 2, 5037 AB Tilburg, The Netherlands; 2 Department of Sociology, Utrecht University, Utrecht, The Netherlands

**Correction** **:** **Psychonomic Bulletin & Review (2023) 30:1609–1620**

10.3758/s13423-022-02235-5

In the abstract of this paper, in the last sentence, the word *overestimate* should have been *underestimate*. So the correct final sentence of the abstract is: “Our studies demonstrate that many psychology researchers underestimate the evidence in favor of a theory if one or more results from a set of replication studies are statistically significant, highlighting the need for better statistical education.”

